# Effect of Docosahexaenoic Acid on Ca^2+^ Signaling Pathways in Cerulein-Treated Pancreatic Acinar Cells, Determined by RNA-Sequencing Analysis

**DOI:** 10.3390/nu11071445

**Published:** 2019-06-26

**Authors:** Suhn Hyung Kim, Yeeun Park, Joo Weon Lim, Hyeyoung Kim

**Affiliations:** Department of Food and Nutrition, Brain Korea 21 PLUS Project, College of Human Ecology, Yonsei University, Seoul 03722, Korea

**Keywords:** calcium, cerulein, docosahexaenoic acid, pancreatic acinar cells

## Abstract

Intracellular Ca^2+^ homeostasis is commonly disrupted in acute pancreatitis. Sustained Ca^2+^ release from internal stores in pancreatic acinar cells (PACs), mediated by inositol triphosphate receptor (IP3R) and the ryanodine receptor (RyR), plays a key role in the initiation and propagation of acute pancreatitis. Pancreatitis induced by cerulein, an analogue of cholecystokinin, causes premature activation of digestive enzymes and enhanced accumulation of cytokines and Ca^2+^ in the pancreas and, as such, it is a good model of acute pancreatitis. High concentrations of the omega-3 fatty acid docosahexaenoic acid (DHA) inhibit inflammatory signaling pathways and cytokine expression in PACs treated with cerulein. In the present study, we determined the effect of DHA on key regulators of Ca^2+^ signaling in cerulein-treated pancreatic acinar AR42 J cells. The results of RNA-Sequencing (RNA-Seq) analysis showed that cerulein up-regulates the expression of IP3R1 and RyR2 genes, and that pretreatment with DHA blocks these effects. The results of real-time PCR confirmed that DHA inhibits cerulein-induced IP3R1 and RyR2 gene expression, and demonstrated that DHA pre-treatment decreases the expression of the Relb gene, which encodes a component of the nuclear factor kappa-light-chain-enhancer of activated B cells (NF-κB) transcriptional activator complex, and the c-fos gene, which encodes a component of activator protein-1 (AP-1) transcriptional activator complex. Taken together, DHA inhibits mRNA expression of IP3R1, RyR2, Relb, and c-fos, which is related to Ca^2+^ network in cerulein-stimulated PACs.

## 1. Introduction

Acute pancreatitis is an inflammatory disease that results in organ dysfunction. This disease is caused by abnormal activation and release of digestive enzymes and it is characterized by increased cytokine release and oxidative stress [1]. Elevated levels of inflammatory cytokines and free radical products are commonly observed in animal models of acute pancreatitis as well as in individuals inflicted by the disease [2,3]. It has been previously proposed that the excessive rise in the intracellular Ca^2+^ concentration ([Ca^2+^]i) observed in pancreatic acinar cells (PACs), the functional units of the exocrine pancreas, triggers the onset of acute pancreatitis [4]. The disruption of calcium signaling in PACs that occurs during acute pancreatitis is well characterized [5,6].

The gastrointestinal peptide cholecystokinin (CCK) causes the release of pancreatic digestive enzymes and the growth of the normal pancreas. However, an abnormally high level of plasma CCK has been reported to occur in patients with chronic pancreatitis [7] and acute pancreatitis [8]. When cholecystokinin (CCK) binds to the CCK receptor, which is a G protein-coupled receptor, heterotrimeric G proteins are activated. Consequently, phospholipase C is activated to generate inositol triphosphate (IP3) and diacylglycerol (DAG), which in turn mediate release of Ca^2+^ from intracellular stores and activation of protein kinase C (PKC) [9]. Ca^2+^ oscillation, generated by the IP3-IP3 receptor (IP3R) and the Ca^2+^-dependent activation of PKC, activates Ca^2+^-relevant signals in PACs [10]. 

Calcium homeostasis in PACs is achieved through coordination of Ca^2+^ release from internal stores, Ca^2+^ entry, and Ca^2+^ extrusion. IP3R and the ryanodine receptor (RyR) are the major calcium release channels, whereas the transient receptor potential 3 (TRPC3) channel and the stromal interaction molecules (STIM)1-Orai1 complex are calcium release-activated calcium channels (CRAC channels) responsible for Ca^2+^ entry into cells. The Ca^2+^-ATPase pump is responsible for calcium clearance [11].

Several studies have highlighted the importance of IP3R and RyR-mediated Ca^2+^ release in the pathophysiology of acute pancreatitis [12,13,14]. IP3Rs and RyRs are sensitive to ROS because they possess reactive cysteine residues that are prone to oxidation. Oxidation of cysteine thiol groups on IP3s and RyRs stimulates the activity of Ca^2+^ release channels, thereby inducing Ca^2+^ release from internal stores [15]. Prolonged Ca^2+^ release promotes a sustained elevation of [Ca^2+^]i through activation of CRAC channels, a process known as store-operated Ca^2+^ entry. Sustained global elevation of [Ca^2+^]i eventually results in zymogen activation, inflammatory injury, and acinar cell death [14,16].

The pancreatic acinar cell is a highly specialized structure developed for synthesis, storage, and secretion of digestive enzymes. The acinar cell is tightly polarized. Its apical pole is densely packed with zymogen granules that secrete digestive enzymes by exocytosis [17]. Since IP3R is located in the apical region of the acinar cells, the IP3-IP3R-induced Ca^2+^ oscillation mediates the start of the Ca^2+^ wave at the apical trigger zone [18]. RyR is diffusely distributed in the basolateral region and is responsible for the early zymogen activation that occurs in the basal compartment [19]. RyR, first identified in the sarcoplasmic reticulum (SR), provokes calcium-induced calcium release, thereby propagating the Ca^2+^ wave [20]. Thus, the general Ca^2+^ signal starts at the trigger zone via IP3R-mediated Ca^2+^ release, and the basolateral activation of RyR, which propagates the Ca^2+^ wave through the Ca^2+^-induced Ca^2+^ release mechanism [21]. 

Cerulein, a CCK analog, induces pancreatitis, which is widely used as an experimental model of acute pancreatitis. Cerulein, at supramaximal doses, induces dysregulation of digestive enzymes, death of acinar cells, and infiltration of inflammatory cells into the rat pancreas [22]. It has been shown that intracellular Ca^2+^ oscillation mediates activation of inflammatory transcription factors and cytokine production [23]. Rapid mobilization of Ca^2+^ from the internal stores is a prominent feature of the inflammatory response associated with several diseases [24]. Studies with experimental models of inflammatory pathology showed that blocking Ca^2+^ signaling significantly attenuates activation of nuclear factor kappa-light-chain-enhancer of activated B cells (NF-κB) and mitogen-activated protein kinase (MAPK) signaling, as well as pro-inflammatory cytokine production [25,26]. One such study showed that ultrafine particles induce NF-κB and activator protein-1 (AP-1) activation, as well as cytokine expression in macrophages, by modulating intracellular calcium [27]. Thus, intracellular Ca^2+^ oscillation may mediate activation of inflammatory transcription factors and cytokine production in cerulein-stimulated PACs. 

Previous investigations revealed that docosahexaenoic acid (DHA) exhibits anti-oxidative and anti-inflammatory properties in various disease models [28]. We have reported that DHA activated peroxisome proliferator-activated receptor γ (PPARγ) and induced catalase to reduce ROS-mediated expression of cytokines in PACs [29]. Cerulein activated the reduced nicotinamide adenine dinucleotide phosphate (NADPH) oxidase and produced cellular ROS, which was suppressed by DHA treatment in PACs [30,31]. Because high levels of CCK lead to high concentrations of intracellular Ca^2+^, DHA inhibition of ROS production might be mediated through its impact on intracellular Ca^2+^.

DHA has also been shown to be effective in alleviating inflammation, as it suppresses inflammatory signaling mediators and reduces cytokine production. DHA inhibits expression of genes encoding NF-κB and its downstream signaling partners cyclooxygenase-2 (COX-2), interleukin (IL)-6, and IL-1β [32,33]. In cerulein-induced acute pancreatitis, DHA suppresses IL-1β and IL-6 gene expression by inhibiting AP-1 activation [34]. 

Therefore, we hypothesize that DHA inhibits Ca^2+^ signaling and its related signaling pathways in cerulein-stimulated rat pancreatic acinar AR42J cells. To identify the regulators involved in cerulein-mediated changes in Ca^2+^ signaling, and to examine the effect of DHA treatment on cerulein-induced alterations in Ca^2+^ signaling and related pathways, we carried out RNA-Sequencing (RNA-Seq) analysis on the AR42J cells. 

## 2. Materials and Methods 

### 2.1. Cell Line and Treatments

Rat pancreatic acinar AR42J cells (CRL 1492) were obtained from the American Type Culture Collection (Manassas, VA, USA) and cultured in Dulbecco’s modified Eagle’s medium (Sigma, St. Louis, MO, USA) supplemented with 10% FBS (Gibco-BRL, Grand Island, NY, USA), 100 U/mL penicillin, and 100 μg/mL streptomycin. 

DHA was dissolved in ethanol (0.5 M solution) (Sigma, St. Louis, MO, USA) and cerulein was dissolved in PBS containing 0.1% BSA (10^−4^ M) [29]. AR42J cells (1 × 10⁵/mL) were treated with a vehicle 0.5 M ethanol (designated as ‘none’) or DHA (50 μM) dissolved in 0.5 M ethanol (designated as ‘DHA’) for 6 h. For cerulein treatment, the cells were pretreated with 0.5 M ethanol (designated as ‘cerulein’) or DHA (50 μM) dissolved in 0.5 M ethanol for 2 h and then stimulated with cerulein (10^−^⁸ M) (designated as ‘cerulein + DHA’) for 4 h. The indicated dose and incubation time of DHA were chosen based on our previous study reporting anti-oxidative and anti-inflammatory effects of DHA on cerulein-stimulated AR42J cells [29]. Further, 50 μM of DHA alone used in the present study had no effect on cell response such as cytokine expression and inflammatory signaling in AR42J cells [29] and cell viability in pancreatic cancer PANC-1 cells for 24 h culture [35].

### 2.2. Preparation of Total RNA Extracts and Library Construction

Total RNA was extracted from cells harvested from culture dishes using TRI reagent (Molecular Research Center, Cincinnati, OH, USA) and then purified using the RNeasy MinElute Cleanup Kit (Quiagen, Valencia, CA, USA) according to the manufacturer’s instructions. The quality of the total isolated RNA was determined by measuring the concentration of total RNA in the extracts from three replicates, using NanoQuant Infinite M200 instrument (Tecan, Männedorf, Switzerland). The total RNA from each sample was pooled for RNA-Seq library construction using TruSeq RNA Sample Prep Kit (Illumina, San Diego, CA, USA). Briefly, cDNA libraries were prepared by purification from 1 μg total RNA, random fragmentation, and reverse transcription. 

### 2.3. RNA-Sequencing and Bioinformatics Analysis

The total RNA library was subjected to transcriptome sequencing. The sequencing was carried out with Macrogen (www.macrogen.co.kr; Seoul, Korea). CLRNASeqTM software (Chunlab, www.chunlab.com; Seoul, Korea) was used for the initial data processing. Raw RNA-Seq reads were trimmed with a quality cutoff of Q30 and the gene expressions were quantified using Cufflinks (https://omictools.com/cufflinks-tool). The RNA-Seq data were normalized by using the relative log expression (RLE) value.

Changes in gene expression of the cells with neither DHA treatment nor cerulein stimulation (treated with 0.5 M ethanol vehicle only) (none), those treated with cerulein, those with DHA treatment alone, and those pretreated with DHA and treated with cerulein (cerulein + DHA) were compared. Differential gene expression analysis was performed using the RNA-Seq analysis module of the CLRNASeq software, with a cutoff set at gene expression of >100 and *p*-value of <0.05. Transcripts with fold change of >1.5 were included as differentially expressed genes (DEGs). After identifying the DEGs, gene ontology (GO) analysis was performed using the DAVID bioinformatics program (https://david.ncifcrf.gov) for gene identification and annotation. The annotation results were categorized under biological process, molecular function, and cellular function. To identify the functional groups and molecular pathways associated with the observed DEGs, the RNA-Seq data were further analyzed using the Kyoto Encyclopedia of Genes and Genomes (KEGG) database (www.genome.jp).

### 2.4. Validation of RNA-Seq Profiles by Real-time Polymerase Chain Reaction (PCR)

Real-time PCR was performed to validate the results generated by the RNA-Seq analysis. Candidate genes were selected in relation to the functional pathway of interest. Total RNA was isolated by using TRI reagent (Molecular Research Center, Inc., Cincinnati, OH, USA). The total RNA was converted to cDNA by treatment with a random hexamer and MuLV reverse transcriptase (Promega, Madison, WI, USA) at 23 °C for 10 min, 37 °C for 60 min, and 95 °C for 5 min. The cDNA was used for real-time PCR with primers specific for rat. The sequences of the primers used are 5’- GAATCAGTGAGTTACTGGGCATGG -3’ (forward) and 5’- CTGGTCTCTGAGTTCTCCAAAAGC-3’(reverse) for RyR2, 5’-TCCCTGGTCAGCAGTGACTC-3’(forward) and 5’-CTCATTTGCTTAGGCTGGCT-3’(reverse) for IP3R1, 5’-AGCGAAAGCGGGGACTGC-3’(forward) and 5’-GATGGTGGGAGGAACAGG-3’(reverse) for Relb, and 5’-GTAGAGCAGCTATCTCCTGA-3’ (forward) 5’-AACGCAGACTTCTCGTCTTC-3’(reverse) for c-fos, and 5’-ACCAACTGGGACGATATGGAG-3’(forward) and 5’-GTCAGGATCTTCATGAGGTAGTC-3’(reverse) for β-actin. For PCR amplification, the cDNA was amplified by 35 repeat cycles with denaturation at 95 °C for 30 s, annealing at 51 °C–55 °C for 30 s, and extension at 72 °C for 30 s. During the first cycle, the 95 °C step was extended to 3 min. The β-actin gene was amplified in the same reaction to serve as the reference gene.

### 2.5. Statistical Analysis

Statistical analysis was performed using the edgeR test and the one-way ANOVA technique. The results were expressed as the mean ± S.E. of three independent experiments. A *p*-value of 0.05 or less was considered statistically significant.

## 3. Results

### 3.1. DHA Inhibits Cerulein-Induced Changes in the Transcriptomic Profile of AR42J Cells 

**Figure 1:**
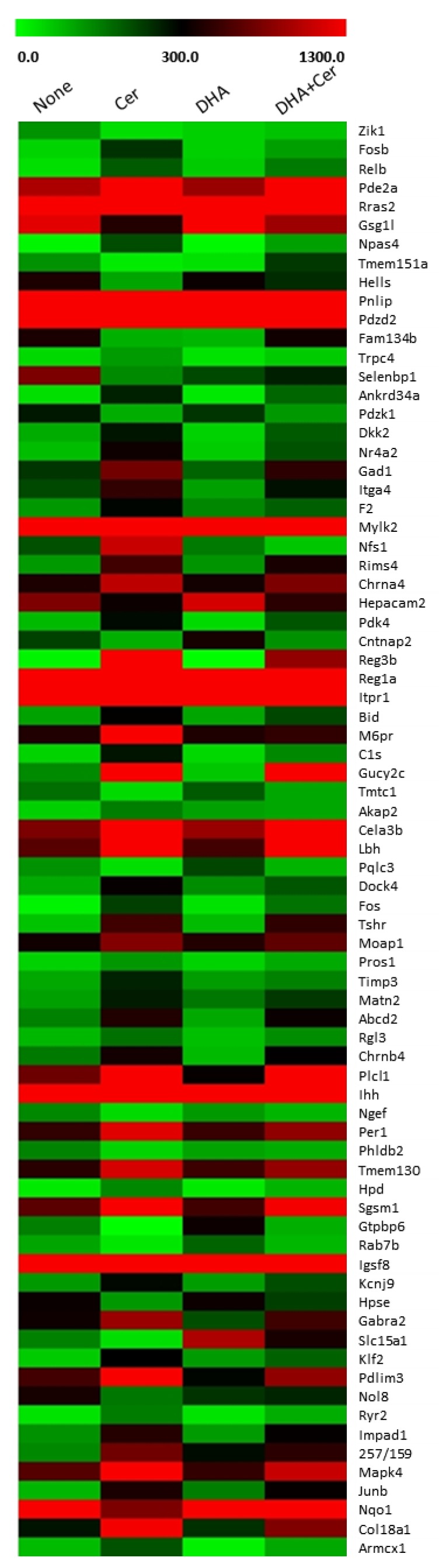
Heatmap of differentially expressed genes (DEGs) that are significantly altered by cerulein treatment, or by pretreatment with docosahexaenoic acid (DHA) followed by cerulein treatment. AR42J cells were treated with a vehicle (designated as ‘none’) or DHA (50 μM) dissolved in vehicle (designated as ‘DHA’) for 6 h. For cerulein treatment, the cells were pretreated with a vehicle (designated as ‘cerulein’) or DHA (50 μM) dissolved in vehicle for 2 h and then stimulated with cerulein (10^−^⁸ M) (designated as ‘cerulein + DHA’) for 4 h. Vehicle is 0.5 M ethanol. Heatmap was drawn based on normalized read counts represented as FPKM (fragment per kilobase of transcript per million mapped reads).

In order to identify substances modulated by cerulein and to examine the effect of DHA on cerulein-induced transcriptomic alterations, RNA-Seq analysis was performed. RNA-Seq analysis identified 339 genes with differential expression between non-stimulated and cerulein-stimulated cells, 181 genes differentially expressed between vehicle-treated and DHA-treated cells, and 116 differentially expressed genes between cerulein-stimulated cells and DHA-treated and cerulein-stimulated cells. Among these genes, 76 genes showed significant differential expression between non-stimulated, cerulein-stimulated, and DHA-treated and cerulein-stimulated cells, with gene expression levels of >100, fold changes of >1.5, and *p* values of <0.05. The heatmap of the filtered DEGs is shown in Figure 1. Out of the significant DEGs, four genes showed differential expression following DHA treatment compared to non-treated cells: Pqlc3 known as a protein coding gene, Gtpbp6 whose function is not yet known, Slc15a1 required for transport of peptides across membranes, and an antioxidant enzyme gene NQO1. 

Genes whose expressions were significantly increased by cerulein were chosen for further computational analysis. The list of DEGs that were up-regulated 1.5-fold or more by treatment with cerulein, and down-regulated 1.5-fold or more by pretreatment with DHA followed by treatment with cerulein is given in Table 1. DHA alone without cerulein stimulation had no significant effect on the expression of the listed genes shown in Table 1. 

### 3.2. DHA Suppresses Cerulein-Induced Alteration in the Calcium Signaling Pathway, Determined by Functional Annotation and Pathway Analysis of DEGs 

The DEGs listed in Table 1 were used as inputs to perform GO term analysis within the DAVID bioinformatics tool suite. The annotation results were categorized under biological process, molecular function, and cellular function. Gene annotation analysis revealed that the DEGs in the cells treated with cerulein are mostly annotated ‘biological process’ among the three GO categories. The common GO terms were metabolic pathways, oxytocin signaling pathway, pancreatic secretion, regulation of actin cytoskeleton, MAPK signaling pathway, cAMP signaling pathway, calcium signaling pathway, and apoptosis. The functions of the impacted pathways and the names of the key pathway genes are listed in Table 2.

**Figure 2:**
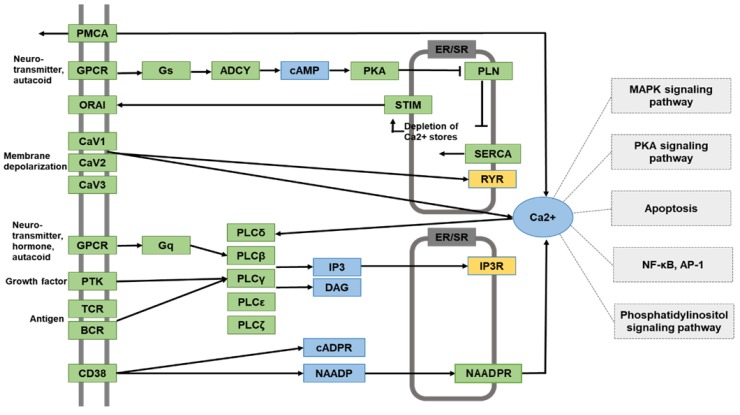
A schematic of the Ca^2+^ signaling network derived from Kyoto Encyclopedia of Genes and Genomes (KEGG). The filtered DEGs identified by the RNA-Seq analysis are colored in yellow boxes (ryanodine receptor (RYR), inositol triphosphate receptor (IP3R)). The molecular pathways associated with Ca^2+^ signaling are colored in light grey boxes (mitogen-activated protein kinase (MAPK), protein kinase A (PKA), Apoptosis, nuclear factor kappa-light-chain-enhancer of activated B cells (NF-κB), activator protein-1 (AP-1), phosphatidylinositol signaling pathways). The molecules in blue are second messengers in signaling transduction pathways.

Next, KEGG pathway analysis was performed to identify the physiological pathways in AR42J cells treated with cerulein. DEGs listed in Table 1, whose expression level was increased by cerulein but decreased by DHA, were assessed. Among these, we focused on Ca^2+^ signaling pathway. The expression of the respective genes encoding the calcium signaling mediators RyR2 and IP3R1 gene expression was notably up-regulated in cells treated with cerulein, but down-regulated in cells pre-treated with DHA prior to exposure to cerulein. KEGG pathway analysis revealed that the effect of DHA on cerulein-stimulated PACs may involve alteration of several signaling pathways that are downstream of calcium release from the ER, such as the MAPK pathway, protein kinase A (PKA) pathway, nuclear factor kappa-light-chain-enhancer of activated B cells (NF-κB) pathway, and AP-1 pathway (Figure 2). Mylk2 is involved in Ca2+ signaling pathway according to the KEGG Mapper, but it has its main role in vascular contraction that is different from pancreatic acinar cell function. Thus, some genes including Myk2, whose functions are unrelated to pancreatic function, were not included in Figure 2. 

### 3.3. DHA Inhibits Expression of RyR2, IP3RI, Relb, and c-fos Genes in Cerulein-Treated AR42J Cells, Determined by Real-Time PCR

**Figure 3:**
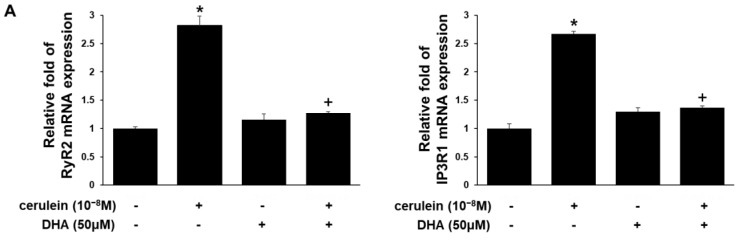

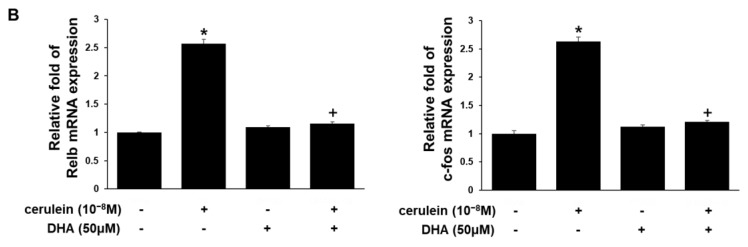
A chart of mRNA expression levels of the RyR2, IP3R1 **(A),** Relb, and c-fos **(B)** in AR42J cells. AR42J cells were treated with a vehicle or DHA (50 μM) dissolved in vehicle for 6 h. For cerulein treatment, the cells were pretreated with a vehicle or DHA (50 μM) dissolved in vehicle for 2 h and then stimulated with cerulein (10^−^⁸ M) for 4 h. Vehicle is 0.5 M ethanol. The mRNA levels were determined by real-time PCR analysis and normalized to the expression of the β-actin gene. *p < 0.05 vs. cells without treatment (cerulein-, DHA - ); +*p* < 0.05 vs. cells with cerulein treatment alone (cerulein +, DHA -). mRNA expression of the cells without treatment (cerulin-, DHA - ) was set at 1. Relative fold of each group was compared to that of the cells without treatment (cerulin-, DHA - ).

In order to validate the inhibitory effect of DHA on the cerulein-induced alterations identified by RNA-Seq analysis, several candidate genes were selected from those assigned to the calcium signaling pathway and subjected to real-time PCR confirmation. The results are in agreement with the RNA-Seq results. Cerulein induced expression of the genes RyR2 and IP3R1 by up to 3-fold, which was suppressed by pretreatment of DHA (Figure 3A). Cerulein increased expression of the Relb gene, a member of the NF-κB gene family, and c-fos gene, which encodes a component of the AP-1 complex (Figure 3B). DHA inhibited cerulein-induced expression of the Relb and c-fos genes (Figure 3B). DHA treatment alone did not affect mRNA expression of RyR2, IP3R1, RelB, and c-fos in PACs (Figure 3). 

## 4. Discussion 

Acute pancreatitis occurs upon autodigestion of the pancreas by digestive enzymes and the induction of the inflammatory response. Disturbances in calcium homeostasis is fundamental to the pathophysiology of acute pancreatitis [36,37]. Calcium homeostasis mediators IP3Rs and RyRs have long been implicated in acute pancreatitis. Inhibitors of IP3Rs and RyRs have proven to be effective in reducing zymogen activation, acinar cell death, and proinflammatory cytokine generation in experimental acute pancreatitis [38,39]. IP3Rs and RyRs trigger the initial release of Ca^2+^ from internal stores such as the ER, and supraphysiological stimulation results in a persistent increase in Ca^2+^ release. This leads to depletion of the internal Ca^2+^ stores, which in turn activates the CRAC channels.

Compared to other types, PACs are relatively ineffective in maintaining low intracellular Ca^2+^ extrusion and are especially mal-equipped for [Ca^2+^]i clearance [36,40]. In the absence of voltage-gated Ca^2+^ channels, calcium signaling primarily depends on internal Ca^2+^ stores [41]. Therefore, PACs are particularly vulnerable to the elevation in intracellular Ca^2+^ spikes. 

In the present study, RNA-Seq analysis of cerulein-treated AR42J cells suggests that cerulein may increase the level of intracellular calcium by up-regulating genes encoding the calcium release-mediating channels, IP3R1 and RYR2. Pretreatment of cells with DHA decreases the expression of the IP3R1 and RYR2 genes up-regulated by cerulein. Therefore, DHA may normalize the cerulein-induced abnormal Ca^2+^ wave in AR42J cells. Functional analysis of molecular pathways suggests that cerulein, via transcriptional regulation of Ca^2+^ release channels, may modulate signaling regulators related to the MAPK, NF-κB, and AP-1 pathways, which may be suppressed by DHA in PACs. 

Experimental validation using real time-PCR confirmed the effect of DHA in reducing cerulein-induced increased expression of the IP3R1 and RYR2 genes. The results obtained from real time-PCR analysis also demonstrate that cerulein increases expression of genes encoding the transcription factors NF-κB and AP-1, and that DHA reduces cerulein-induced expression of the Relb gene, which encodes a component of the NF-κB complex, and the c-fos gene, which encodes a component of the AP-1 complex.

Several studies have suggested that a link exists between the prolonged increase in [Ca^2+^]i and up-regulation of the genes encoding NF-κB and AP-1. Elevated [Ca^2+^]i and Ca^2+^-dependent activation of PKC may stimulate activation of AP-1 and NF-κB. The causal association of NF-κB activation and Ca^2+^ signaling is well known [42]. Stimulation with high-dose cerulein activates NF-κB in the pancreatic acini via increased [Ca^2+^]i and activation of PKC [43]. Calcium blockers and chelators abrogate cerulein-induced NF-κB activation and IκB degradation in PACs [44].

Abnormal Ca^2+^ signaling is also indicated to be relevant to AP-1 activation [45]. Carbachol, a secretagogue similar to CCK, increases expression of the genes c-fos and c-jun, which encode AP-1. The increased expression is mediated by changes in [Ca^2+^]i, calmodulin, PKC, and MAPK [46]. Ramnath et al. [47] demonstrated that the rise in [Ca^2+^]i and Ca^2+^-dependent activation of PKC mediates AP-1 activation and subsequent chemokine production in substance P-induced experimental acute pancreatitis. Chelating cytosolic calcium, inhibition of PLC, and inhibition of Ca^2+^-dependent PKC each block substance P-induced [Ca^2+^]i elevation, activation of AP-1, and chemokine production in PACs. 

DHA has been suggested to positively modulate Ca^2+^ signaling in the ER [48]. Begum et al. [49] revealed that DHA inhibits excess Ca^2+^ release from the ER and further represses store-operated Ca^2+^ overload in astrocytes, specifically by blocking IP3R. In cardiac myocytes, DHA significantly suppresses the Ca^2+^ spark from SR, and inhibited RyR activity [50,51].

DHA treatment also appears to be effective in suppressing inflammatory mediators. DHA decreases production of pro-inflammatory cytokines, such as TNF-α, IL-6, and IL-1β, by inhibiting phosphorylation of NF-κB subunit p65 in bovine mammary epithelial [52] and cardiac cells [53]. In ischemic rats, DHA decreases the level of pro-inflammatory cytokines and chemokines, and inhibits phosphorylation of c-jun and AP-1 DNA binding activity [54].

As shown in Table 2, c-fos is involved in cAMP signaling and MAPK signaling, while Relb is relevant to MAPK signaling. c-Fos gene contains a Ca^2+^/cAMP response element (Ca^2+^/CRE), and its expression and post-translational modification is, in part, affected by Ca^2+^ and cAMP level [55]. The elevation of cAMP further enhances expression of c-fos in cardiac myocytes [56] and myeloid leukemia cells [57]. c-Fos also possesses a serum response element (SRE), and MAPK activation induces c-fos expression via SRE [58,59]. Moreover, Relb is a member of the NF-κB family, which demands synergistic activation of MAPK signaling pathways for symptomatic and pathologic inflammatory response [60,61]. Therefore, cerulein-induced increase in c-fos and Relb may be related to activation of cAMP-PKA pathway, MAPK pathway, NF-κB, and AP-1 as shown in Figure 2. Thus, the inhibitory effect of DHA on Ca^2+^ signaling may also be associated with suppression of PKA, MAPK, NF-κB, and AP-1 pathways. Further studies should be performed to assess the effects of DHA on PKA, MAPK, NF-κB, and AP-1 pathways in cerulein-stimulated PACs.

The main findings of this study are that DHA inhibits mRNA expression of IP3R1, RyR2, Relb, and c-fos, which is related to Ca^2+^ signaling in cerulein-stimulated PACs. Further studies should be performed exploring whether DHA affects RyR, IP3R, Relb, and c-FOS at post-transcriptional level and their functional roles involving the calcium network to determine the effect of DHA on cerulein-induced acute pancreatitis. 

## 5. Conclusions

DHA inhibits mRNA expression of IP3R1 and RyR2 which are upregulated in cerulein-stimulated PACs. Cerulein increases expression of the Relb gene, a member of the NF-κB gene family, and c-fos gene, a component of the AP-1 complex. DHA inhibits cerulein-induced expression of the Relb and c-fos genes in PACs. Since IP3R1, RyR2, Relb, and c-fos are involved in Ca^2+^ network, DHA may inhibit Ca^2+^ signaling by suppressing key regulators in cerulein-treated PACs.

## Figures and Tables

**Table 1:** Genes whose expressions were changed by treatment of cerulein or DHA or by pretreatment with DHA followed by cerulein treatment.

Gene Name	None Mapped Read ^1^	Cerulein Mapped Read ^1^	DHA Mapped Read ^1^	Cerulein+DHA Mapped Read ^1^
Fosb	49	242	55	113
Relb	38	194	62	156
Pde2a	997	2143	919	1402
Rras2	2058	4369	1837	3879
Npas4	10	210	7	112
Pnlip	3165	9804	2821	6068
Pdzd2	35	124	47	55
Trpc4	44	117	32	62
Ankrd34a	38	261	26	178
Dkk2	97	275	53	194
Nr4a2	77	363	60	204
Gad1	237	763	182	483
Itga4	214	500	112	280
F2	121	293	140	188
Mylk2	1651	4457	1293	2648
Nfs1	205	1109	156	67
Rims4	118	567	125	401
Chrna4	414	1059	381	803
Pdk4	81	287	43	198
Reg3b	12	2873	2	892
Reg1a	11,048	26,879	9104	12,476
Itpr1	2398	6178	2656	4547
Bid	109	317	105	213
M6pr	421	1352	426	485
C1s	51	276	45	139
Gucy2c	138	2830	65	1420
Akap2	54	151	110	102
Cela3b	810	5573	915	3610
Lbh	652	2070	572	1366
Dock4	99	331	135	198
Fos	14	226	34	164
Tshr	70	556	80	496
Moap1	372	832	444	684
Pros1	54	122	52	100
Timp3	102	260	116	146
Matn2	111	268	159	234
Abcd2	146	436	101	346
Rgl3	84	168	76	133
Chrnb4	157	382	81	312
Plcl1	738	1457	332	1357
Ihh	3020	10,806	1511	6831
Per1	146	436	101	346
Tmem130	465	1162	550	899
Hpd	24	139	24	88
Sgsm1	666	1706	560	1286
Igsf8	2961	9669	3829	7782
Kcnj9	119	290	115	209
Gabra2	361	922	209	558
Klf2	61	302	117	185
Pdlim3	574	1796	294	886
RyR2	32	154	32	96
Impad1	129	447	117	326
Egr1	138	752	286	478
Mapk4	645	1750	508	1098
Junb	32	154	32	96
Col18a1	277	1286	243	818
Armcx1	80	203	17	102

AR42J cells were treated with a vehicle (designated as ‘none’) or DHA (50 μM) dissolved in vehicle (designated as ‘DHA’) for 6 h. For cerulein treatment, the cells were pretreated with a vehicle (designated as ‘cerulein’) or DHA (50 μM) dissolved in vehicle for 2 h and then stimulated with cerulein (10^−^⁸ M) (designated as ‘cerulein + DHA’) for 4 h. Vehicle is 0.5 M ethanol. ^1^ Raw reads of 200–400 bp fragments from each sample library were obtained and aligned to reference genome transcripts. The mapped read counts were normalized by RLE value to quantify relative abundance of each gene.

**Table 2:** A list of the functions and key genes of the corresponding physiological pathways impacted by cerulein in AR42J cells.

Metabolic Pathways	Gad1, Hpd, Hpse, Impad1, Nfs1, Pnlip
Neuroactive ligand-receptor interaction	Chrna4, Chrnb4, F2, Gabra2, Tshr
Oxytocin signaling pathway	Fos, Itpr1, Kcnj9, Mylk2, Ryr2
Apelin signaling pathway	Itpr1, Klf2, Mylk2, Rras2, Ryr2
Proteoglycans in cancer	Hpse, Ihh, Itpr1, Rras2, Timp3
Pancreatic secretion	Cela3b, Itpr1, Pnlip, Ryr2
Regulation of actin cytoskeleton	F2, Itga4, Mylk2, Rras2
cAMP signaling pathway	Fos, Rras2, Ryr2, Tshr
MAPK signaling pathway	Fos, Nr4a1, Relb, Rras2
Calcium signaling pathway	Itpr1, Mylk2, Ryr2
cGMP-PKG signaling pathway	Itpr1, Mylk2, Pde2a
Apoptosis	Bid, Fos, Itpr1
Autophagy	Itpr1, Rab7b, Rras2

cAMP, cyclic adenosine monophosphate; MAPK, mitogen-activated protein kinase; cGMP-PKG; cyclic guanosine monophosphate-protein kinase G.
